# Synthesis and Analgesic Activity of Some New Pyrazoles and Triazoles Bearing a 6,8-Dibromo-2-methylquinazoline Moiety

**DOI:** 10.3390/molecules161210187

**Published:** 2011-12-07

**Authors:** Hosam A. Saad, Nermen A. Osman, Ahmed H. Moustafa

**Affiliations:** 1 Department of Chemistry, Faculty of Science, Taif University, Taif 21974, Saudi Arabia; 2 Department of Pharmaceutical Organic Chemistry, Faculty of Pharmacy, Zagazig University, Zagazig 44511, Egypt; Email: naosman2007@yahoo.com; 3 Department of Chemistry, Faculty of Science, Zagazig University, Zagazig 44511, Egypt; Email: ah_hu_mostafa@yahoo.com

**Keywords:** isoindol-5-one, pyrazole, quinazolin-4(3*H*)-one, thiazole, 1,2,4-triazole, 1,3,4-thiadiazole, analgesic activity

## Abstract

2-(6,8-Dibromo-2-methylquinazolin-4-yloxy)-acetohydrazide (**4**) was prepared by the reaction of 6,8-dibromo-2-methylbenzo-[*d*][1,3]oxazin-4-one with formamide to afford quinazolinone **2**, followed by alkylation with ethyl chloroacetate to give the ester **3**. Treatment of ester **3** with hydrazine hydrate and benzaldehyde afforded **4** and styryl quinazoline **5**. The hydrazide was reacted with triethyl orthoformate, acetylacetone and ethyl acetoacetate and benzaldehyde derivatives to afford the corresponding pyrazoles **6**, **7**, **9** and hydrazone derivatives **10a-c**. Cyclization of hydrazones **10a-c** with thioglycolic acid afforded the thiazole derivatives **11a-c**. Reaction of the hydrazide with isothiocyanate derivatives afforded hydrazinecarbothioamide derivatives **12a-c**, which cyclized to triazole-3-thiols and thiadiazoles **13a-c** and **14a-c**, respectively. Fusion of the hydrazide with phthalimide afforded the annelated compound 1,2,4-triazolo[3,4-*a*]isoindol-5-one (**15**). The newly synthesized compounds were characterized by their spectral (IR, ^1^H-, ^13^C-NMR) data. Selected compounds were screened for analgesic activity.

## 1. Introduction

Quinazolin-4(3*H*)-one and its derivatives are a class of heteroaromatic compounds that have drawn much attention due to their biological and pharmaceutical activities [1,2,3,4,5,6,7,8,9,10,11]. A brief survey on the biological activities of quinazolin-4(3*H*)-one derivatives showed anti-inflammatory [12,13,14], antitumor [15,16,17,18], anti HIV [19], antibacterial [20,21,22], as well as CNS depressant and anticonvulsant activities [23,24]. 4-Substituted quinazolines were also studied as anticancer agents for their strong ability to inhibit several receptor tyrosine kinases [25]. Derivatives of quinazolin-4-one are potential drugs which can possess hypnotic [26], analgesic [27], anthelmintic [28], neuroleptic [29], antiallergic, antimalarial and other effects [30,31]. On the other hand, it was found that not only quinazoline derivatives showed chemotherapeutic activity, but also pyrazole [32], pyrazolone [33], thiadiazoles [34] as well as triazole [35,36] moieties possess this activity. Moreover, the increasing biological importance of quinazolinone derivatives particularly in chemotherapy, promoted us to develop and synthesize the new pyrazolone, pyrazole, thiazolidine, triazole, thiadiazole and triazolo[3,4-*a*]isoindole molecules with a 6,8-dibromoquinazoline substituent moiety, with the aim of obtaining some novel heterocyclic systems with potentially enhanced biological properties.

## 2. Chemistry

**Scheme 1:**
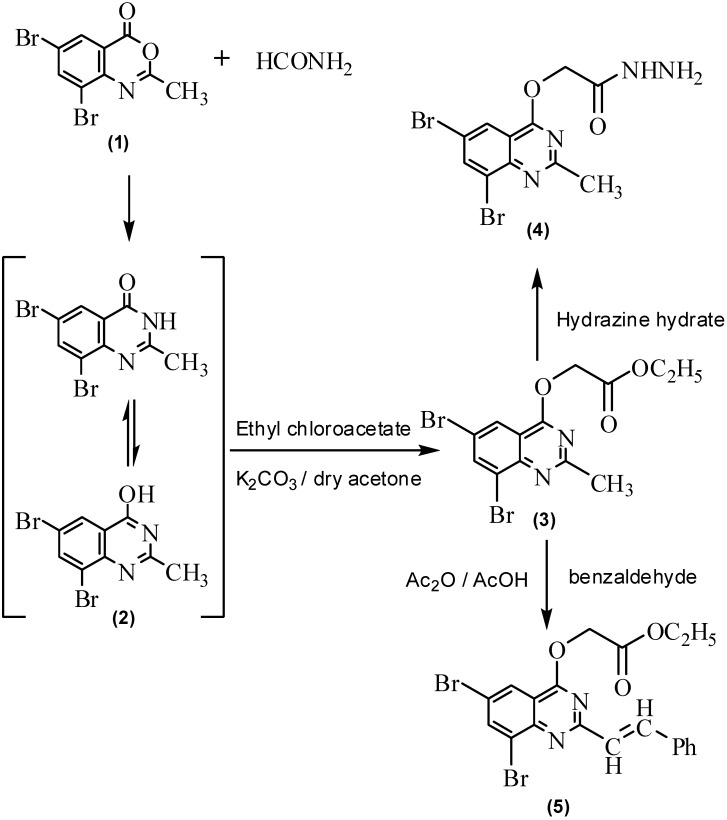
Synthesis and reactions of ethyl 2-(6,8-dibromo-2-methylquinazolin-4-yloxy)-acetate (**3**).

In the view of high biological and pharmacological activity of quinazoline derivatives, we reported in previous work certain substituted quinazoline derivatives [37], so we aimed to continue the previous study in this work. 6,8-Dibromo-2-methyl-4*H*-benzo[*d*][1,3]oxazin-4-one (**1**) was refluxed with formamide to produce 6,8-dibromo-2-methylquinazolin-4(3*H*)one (**2**). Reaction of compound **2** with ethyl chloroacetate in dry acetone in presence of potassium carbonate afforded ethyl 2-(6,8-dibromo-2-methylquinazolin-4-yloxy)acetate (**3**). Hydrazinolysis of compound **3** with hydrazine hydrate gave the corresponding 2-(6,8-dibromo-2-methylquinazolin-4-yloxy)acetohydrazide (**4**), which was used as starting material for preparation of some other quinazoline derivatives (Scheme 1). The IR spectrum of compound **2** showed bands at 3,350, 1,685 and 1,620 cm^−1^ for NH, C=O and C=N. Its ^1^H-NMR spectrum gave signals at δ 2.40, 8.11, 8.21 and 12.30 ppm, characteristic for CH_3_, two aromatic protons and OH groups. IR, ^1^H-, ^13^C-NMR and microanalysis spectral data for compounds **3** and **4** are all in agreement with their assigned structures. The Aldol type condensation of **3** with benzaldehyde afforded ethyl 2-(6,8-dibromo-2-styrylquinazolin-4-yloxy)acetate (**5**, Scheme 1). The ^1^H-NMR spectrum of compound **5** showed signals at 1.30, 4.12 and 4.90 ppm for CH_3_, CH_2_O and OCH_2_CO groups, with disappearance of the CH_3_ signal of the quinazoline ring; in addition, doublet signals at δ 6.95, 7.03 ppm characteristic for a styryl group with coupling constants *J *= 15.98 and 16.01 Hz were observed.

**Scheme 2:**
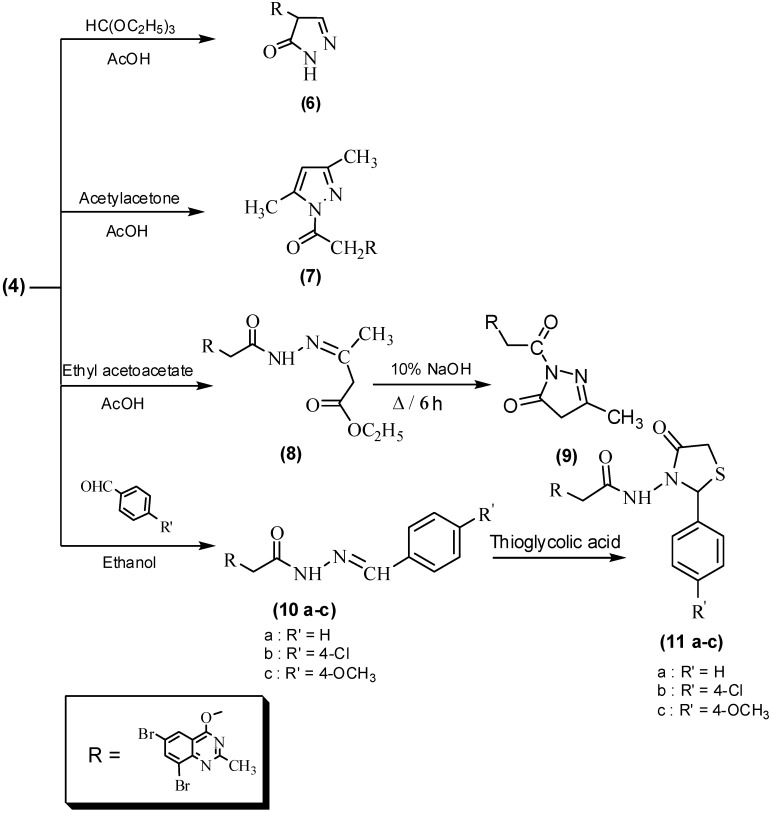
Reactions of 2-(6,8-dibromo-2-methylquinazolin-4-yloxy)acetohydrazide (**4**).

The synthesis of pyrazolone **6** and pyrazole derivatives **7**, as outlined in Scheme 2, involved treating 2-(6,8-dibromo-2-methylquinazolin-4-yloxy)acetohydrazide (**4**) with triethyl orthoformate and acetyl-acetone, respectively. The IR spectrum of compound **6** showed bands at 3,285, 1,685 cm^−1^ for NH and C=O functions, while, its ^1^H-NMR spectrum showed signals at δ 2.57, 4.83 ppm due to CH_3_ and saturated CH in the pyrazolone ring, in addition to a doublet signal at δ 7.50 ppm for the pyrazolone CH=N. The ^13^C-NMR spectrum of compound **6** gave signals at δ 22.4 and 82.2 ppm characteristic for CH_3_ and OCHCO groups. The structure of compound **7** was corroborated by IR, ^1^H-, ^13^C-NMR and elemental analysis. Furthermore, ethyl 3-(2-(2-(6,8-dibromo-2-methylquinazolin-4-yloxy)hydrazono)-butanoate (**8**) was obtained employing the reaction of **4** with ethyl acetoacetate. Compound **8** was intramolecularly cyclized on treatment with 10% sodium hydroxide to yield **9** (Scheme 2).

Treatment of compound **4** with *p-*substituted benzaldehyde in absolute ethanol afforded benzylidine hydrazide derivatives **10a-c**, which were cyclized in the presence of thioglycolic acid to obtain thiazolidine derivatives **11a-c**, respectively (Scheme 2). The structure of compounds **10a-c** was characterized by the presence of bands between 3,280–3,290 and 1,680–1,685 cm^−1^ for the amide groups NH and C=O functions. The ^1^H-NMR spectra of **10a-c** showed a singlet signal at δ 4.95 ppm characteristic for OCH_2_CO, in addition, the important signal at δ 10.2 ppm for the CH=N of the Schiff’s base. The signals in the ^13^C-NMR spectra of compounds **10a-c** confirmed the structures and are mentioned in the Experimental section. Compounds **11a-c** showed in their ^1^H-NMR spectra characteristic signals at δ 2.85–2.95, 3.67–3.69, and 4.89–4.98 ppm attributed to the CH_3_, CH_2_S and CH_2_O groups, respectively. In addition, signals appearing at δ 5.79–5.92 ppm correspond to the CHAr group in the thiazole ring. The IR spectra of compounds **11a-c** showed absorption bands at 3,285–3,310, 1,690–1,695 and 1,680–1,685 cm^−1^ for NH and 2 C=O groups, while the ^13^C-NMR spectrum of compound **11b** showed chemical shift signals at δ 23.3, 39.4, 57.7 and 61.9 ppm characteristic for CH_3_, CH_2_S, NCS and CH_2_O groups which confirmed the thiazole ring formation in **11a-c**.

**Scheme 3:**
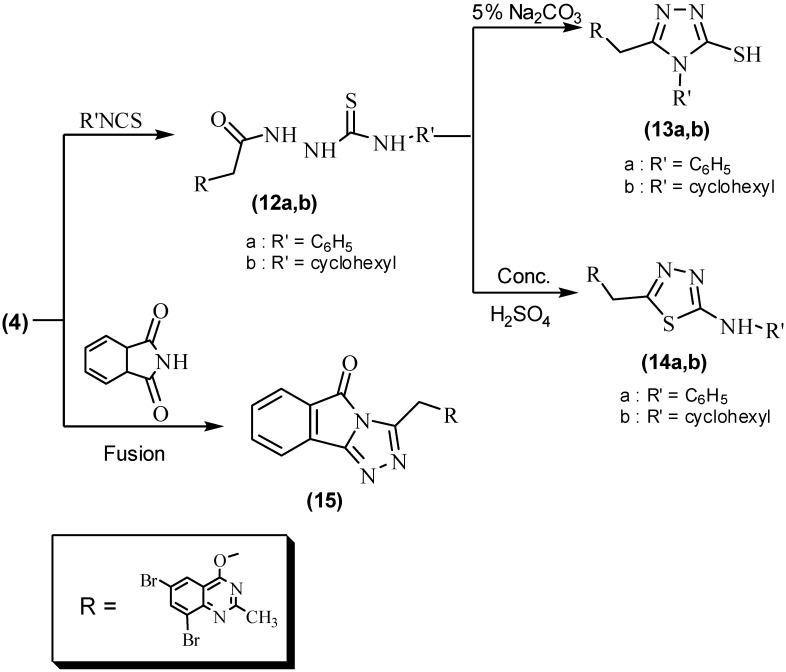
Further reactions of 2-(6,8-dibromo-2-methylquinazolin-4-yloxy)acetohydrazide (**4**).

The new triazole and thiadiazole derivatives **13a,b** and **14a,b** were obtained from the reactions of the starting hydrazide **4**. Reaction of compound **4** with phenyl and cyclohexyl isothiocyanate gave hydrazinecarbothioamide derivatives **12a,b**, which was followed by their cyclization using 5% Na_2_CO_3_ solution to form **13a,b** and with conc. H_2_SO_4_ to give **14a,b**, as reported in the literature [38] (Scheme 3). Compounds **12a,b** were identified by the presence of bands in their IR spectra at 1,235 and 1,220 cm^−1^ for a C=S group. The structures of **12a,b** were also conformed on the basis of their ^1^H-NMR spectra and elemental analysis data. The structures of **13a,b** were deduced from their correct IR, ^1^H-, ^13^C-NMR and elemental analysis data. The ^1^H-NMR spectrum of compound **13a**, for example, showed signals at δ 2.56, 4.95, 13.35 ppm for the CH_3_, CH_2_O and SH groups, while in **13b** a multiplet signal at δ 1.22–1.95 ppm for the cyclohexane ring was seen, in addition to signals at 2.51, 4.95 and 13.38 ppm characteristic for CH_3_, CH_2_O and SH groups. In compounds **14a** and **14b**, the IR spectra gave the absorption bands at 3,280 and 1,618–1620 cm^−1^ for the NH and C=N groups in the thiadiazole ring. The ^1^H-NMR spectrum of compound **14a** showed signals at δ 2.56, 4.01 and 4.85 ppm for the CH_3_, NH and CH_2_O groups, in addition to signals at δ 6.50-8.38 for the seven protons of the aromatic ring. The ^1^H-NMR spectrum of compound **14b** showed signals at δ 1.22–1.95 ppm for the cyclohexane ring, in addition to signals at δ 2.56, 4.08 and 4.98 ppm characteristic for CH_3_, NH and CH_2_O groups. The ^13^C-NMR spectra for **13a,b** and **14a,b** were used to assign the structures. Our research work was finally extended to study the fusion of starting hydrazide **4** with phthalimide to afford 3-[(6,8-dibromo2-methylquinazolin-4-yloxy)methyl]-5*H*[1,2,4]triazolo[3,4-*a*]isoindol-5-one (**15**, Scheme 3).

**Scheme 4:**
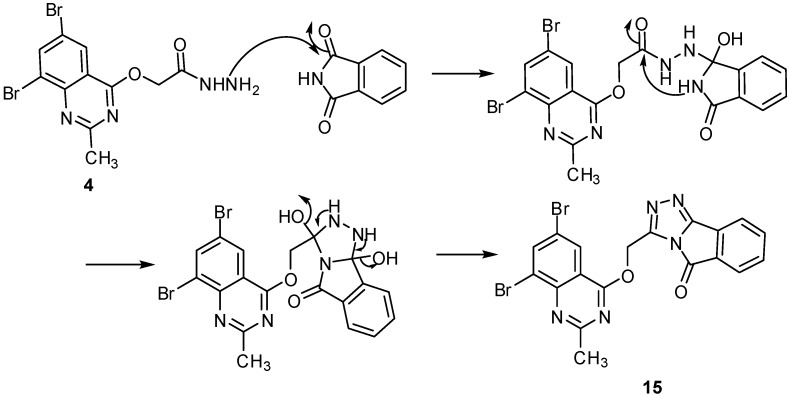
The mechanism of formation of compound **15**.

The IR spectrum of **15** gave absorption bands at 1,686 and 1,618 cm^−1^ for C=O and C=N groups. Its ^1^H-NMR spectrum showed signals at δ 2.57 and 4.98 ppm for the CH_3_ and CH_2_O groups, in addition to a multiplet signal in the aromatic region at δ 7.50–8.35 ppm characteristic of six aromatic protons. Its ^13^C-NMR spectrum showed signals at δ 23.2 and 61.9 ppm for the CH_3_ and CH_2_O groups, in addition to 17 lines in the sp^2^ region which are characteristic for the Ar-C, 4 C=N and C=O groups. The suggested mechanism for formation of compound **15** is given in Scheme 4.

## 3. Pharmacological Studies

### 3.1. Analgesic Activity

Analgesic activity was examined by using the hot-plate test protocol [40,41]. Sixty *Webster* mice of both sexes weighting 20–25 g are used for study. All animals were fed diet in pellets following standard good laboratory practices. Mice were maintained under 12 h light/dark cycles with controlled temperature (22 °C). Experiments were performed in accordance with the standard institutional ethical guidelines. After incubation period (one weak) mice were classified into ten groups. One group as negative control received saline, the second group received vehicle (acacia gum) and the third group received valdecoxib (g) as a reference drug, while the other groups received the nine test compounds by subcutaneous infusion (SC administration). Mice were dropped gently in a dry glass beaker of 1 dm^3^ capacity maintained at 50–50.5 °C. Normal reaction time in seconds for all mice was determined at time intervals of 10, 30, 60 and 120 minutes, this is the interval extending from the instant the mouse reaches the hot beaker till the animals licks its feet or jump out the beaker (dose 5 mg/kg) [39]. The relative potencies to valdecoxib (g) were then determined (Table 1).

**Table 1:** Analgesic activities of some new synthesized compounds.

Comp. NO.	Comparative analgesic potency to Valdecoxib after time in minutes			
	10 min.	30 min.	60 min.	120 min.
6	0.48 ± 0.01	0.52 ± 0.04	0.68 ± 0.06	1.33 ± 0.08
7	0.76 ± 0.03	0.98 ± 0.09	1.23 ± 0.11	2.22 ± 0.20
9	0.44 ± 0.01	0.51 ± 0.05	0.63 ± 0.06	1.29 ± 0.16
11a	0.52 ± 0.01	0.80 ± 0.07	0.87 ± 0.08	1.92 ± 0.08
11c	0.48 ± 0.02	0.59 ± 0.05	0.75 ± 0.07	1.59 ± 0.04
13a	0.65 ± 0.01	0.98 ± 0.08	1.03 ± 0.01	2.25 ± 0.18
13b	0.56 ± 0.01	0.86 ± 0.05	0.98 ± 0.08	2.36 ± 0.21
14a	0.68 ± 0.02	0.88 ± 0.07	1.20 ± 0.14	2.52 ± 0.14
14b	0.60 ± 0.02	0.96 ± 0.03	1.01 ± 0.01	2.35 ± 0.12
Valdecoxib (g)	1.00	1.00	1.00	1.00

All results were significantly different from the standard and normal control. Value at P = 0.05.

### 3.2. Results

All the tested compounds exhibited more potent analgesic activity than valdecoxib(g) as a reference drug (Table 1). Compounds **7**, **13a**, **13b**, **14a** and **14b** showed more than twice the activity of Valdecoxib(g) after two hours. 

## 4. Experimental

Melting points are uncorrected and were recorded in open capillary tubes on a Stuart SMP3 melting point apparatus. Infrared spectra were recorded on a FTIR 1600 spectrophotometer using KBr discs. ^1^H and ^13^C-NMR spectra were measured on an AC 250 MHz spectrometer. All chemical shifts were reported as δ (ppm) scale using TMS as the standard and coupling-constant values are given in Hz. The solvent for NMR spectra was deuterodimethylsulfoxide. The microanalysis results were within ±0.3% of the calculated values. The pharmacological study was carried out at the National Research Center (Center of Excellence for Advanced Sciences, Cancer Biology Research Laboratory).

*6,8-Dibromo-2-methyl-4H-benzo[d][1,3]oxazin-4-one* (**1**). 3,5-Dibromo-2-acetamidobenzoic acid (3.50 g) in acetic anhydride (20 mL) was heated on a water bath for 1.5 h, and then left to cool at room temperature to give a pale yellow powder, which was crystallized from ethanol. Yield 95%; m.p.: 140–142 °C. IR (KBr): 1,730 cm^−1^ (C=O), 1,150 cm^−1^ (C-O, ether); ^1^H-NMR: δ = 2.50 (s, 3H, CH_3_), 8.14, 8.23 (2s, 2H, Ar-H); ^13^C-NMR: δ = 21.7 (CH_3_), 119.1, 121.2, 122.5, 130.3, 142.4, 143.6, 164.1, 172.2 (Ar-C, C=N and C=O). Anal. Calcd for C_9_H_5_Br_2_NO_2_ (318.95): C, 33.89; H, 1.58; N, 4.39. Found: C, 33.88; H, 1.57; N, 4.39.

*6,8-Dibromo-2-methylquinazolin-4(3H)-one* (**2**). Compound **1** (2.0 g) was refluxed in formamide (10 mL) for 2 h, cooled, and then poured onto water to give a pale yellow powder, which was crystallized from ethanol. Yield 75%; m.p.: 298–300 °C. IR (KBr): 3,350 cm^−1^ (NH), 1,685 cm^−1^ (C=O), and 1,620 cm^−1^ (C=N); ^1^H-NMR: δ = 2.40 (s, 3H, CH_3_), 8.11, 8.21 (2s, 2H, Ar-H), 12.3 (br, 1H, NH); ^13^C-NMR: δ = 21.8 (CH_3_), 117.7, 123.4, 127.7, 139.2, 145.7, 146.7, 171.1 and 177.4 (Ar-C, C=N and C=O). Anal. Calcd for C_9_H_6_Br_2_N_2_O (317.96): C, 34.00; H, 1.90; N, 8.81. Found: C, 34.01; H, 1.92; N, 8.79.

*Ethyl 2-(6,8-dibromo-2-methylquinazolin-4-yloxy)acetate* (**3**). A mixture of **2** (10 mmol), ethyl chloroacetate (10 mmol) in dry acetone (20 mL) and potassium carbonate (2.0 g) was refluxed for 6 h, cooled, and poured onto water to afford a pale brown powder that was crystallized from ethanol. Yield 90%; m.p.: 124–126 °C. IR (KBr): 1,730 cm^−1^ (C=O), 1,618 cm^−1^ (C=N) and 1,185 cm^−1^ (C-O, ether); ^1^H-NMR: δ = 1.30 (t, 3H, *J* = 7.0 Hz, CH_3_CH_2_), 2.59 (s, 3H, CH_3_), 4.26 (q, 2H, *J* = 6.98 Hz, CH_2_CH_3_), 4.85 (s, 2H, CH_2_O), 8.11, 8.21 (2s, 2H, Ar-H); ^13^C-NMR: δ = 14.1, 23.5 (2CH_3_), 45.6, 62.3 (2CH_2_O), 119.6, 129.1, 140.6, 144.8, 147.1, 155.0, 166.0 and 167.5 (Ar-C, C=N and C=O). Anal. Calcd for C_13_H_12_Br_2_N_2_O_3_ (404.05): C, 38.64; H, 2.99; N, 6.93. Found: C, 38.63; H, 2.98; N, 6.95.

*2-(6,8-Dibromo-2-methylquinazolin-4-yloxy)acetohydrazide* (**4**). A mixture of **3** (10 mmol) and hydrazine hydrate (20 mmol) in ethanol (20 mL) was refluxed for 4 h. The reaction mixture was concentrated and left to cool to give colorless crystals. Yield 80%; m.p.: 184–186 °C. IR (KBr): 3,280 cm^−1^ (br, NH and NH_2_), 1,675 cm^−1^ (C=O); ^1^H-NMR: δ = 2.59 (s, 3H, CH_3_), 4.64 (s, 2H, NH_2_), 4.98 (s, 2H, CH_2_O), 8.16, 8.35 (2s, 2H, Ar-H), 9.37 (s, 1H, NH); ^13^C-NMR: δ = 23.1 (CH_3_), 61.5 (CH_2_O), 118.6, 128.1, 139.5, 139.8, 143.7, 149.7, 165.8, 167.0 and 176.2 (Ar-C, 2C=N and C=O). Anal. Calcd for C_11_H_10_Br_2_N_4_O_2_ (390.03): C, 33.87; H, 2.58; N, 14.36. Found: C, 33.85; H, 2.59; N, 14.33.

*Ethyl 2-(6,8-Dibromo-2-methylquinazolin-4-yloxy)acetate* (**5**). A mixture of **3** (10 mmol) and benzaldehyde (10 mmol) was refluxed in acetic acid/acetic anhydride (1:1, 20 mL) for 5 h, cooled and the reaction mixture was poured onto cold water to afford a colorless powder which was crystallized from acetic acid. Yield 90%; m.p.: 148–150 °C. IR (KBr): 1,730 cm^−1^ (C=O), 1,620 cm^−1^ (C=N) and 1,180 cm^−1^ (C-O, ether); ^1^H-NMR: δ = 1.30 (t, 3H, *J* = 7.02 Hz, CH_3_CH_2_), 4.12 (q, 2H, *J* = 6.99 Hz, CH_2_CH_3_), 4.90 (s, 2H, CH_2_O), 6.95 (d, 1H, *J* = 15.9 Hz, CH=CH), 7.03 (d, 1H, *J* = 16.0 Hz, CH=CH), 7.33–7.60 (m, 5H, Ar-H), 7.95, 8.22 (2s, 2H, Ar-H). Anal. Calcd for C_20_H_16_Br_2_N_2_O_3_ (492.16): C, 48.81; H, 3.28; N, 5.69. Found: C, 48.83; H, 3.29; N, 5.68.

*4-(6,8-Dibromo-2-methylquinazolin-4-yloxy)-1H-pyrazol-5(4H)-one* (**6**). A mixture of **4** (10 mmol) and triethyl orthoformate (16 mL) with a few drops of acetic acid was refluxed for 2 h. After cooling the reaction mixture was poured onto water. The solid obtained was filtered off, dried and crystallized from ethanol to give colorless crystals. Yield 95%; m.p.: 168–170 °C. IR (KBr): 3,285 cm^−1^ (NH), 1,685 cm^−1^ (C=O) and 1,625 cm^−1^ (C=N); ^1^H-NMR: δ = 2.57 (s, 3H, CH_3_), 4.83 (d, 1H, OCH, pyrazolone), 7.50 (d, 1H, CH=N, pyrazolone), 7.95, 8.30 (2s, 2H, Ar-H), 9.66 (s, 1H, NH); ^13^C-NMR: δ = 23.4 (CH_3_), 82.2 (OCHCO), 119.6, 122.4, 123.1, 129.1, 140.5, 144.1, 155.0, 166.8, 169.0 and 177.2 (Ar-C, 3C=N and C=O). Anal. Calcd for C_12_H_8_Br_2_N_4_O_2_ (400.03): C, 36.03; H, 2.02; N, 14.01. Found: C, 36.02; H, 2.00; N, 14.00.

*2-(6,8-Dibromo-2-methylquinazolin-4-yloxy)-1-(3,5-dimethyl-1H-pyrazol-1-yl)ethanone* (**7**). A mixture of **4** (10 mmol) and acetylacetone (10 mmol) in acetic acid (10 mL) was refluxed for 6 h. After cooling, the reaction mixture was poured onto ice-water. The colorless powder obtained was crystallized from ethanol. Yield 80%; m.p.: 165–166 °C. IR (KBr): 1,680 cm^−1^ (C=O) and 1,618 cm^−1^ (C=N); ^1^H-NMR: δ = 2.56 (s, 3H, CH_3_), 2.78, 2.99 (2s, 6H, 2CH_3_, pyrazole), 4.90 (s, 2H, CH_2_O), 6.65 (s, 1H, pyrazole), 8.11, 8.34 (2s, 2H, Ar-H); ^13^C-NMR: δ = 23.4, 29.4, 45.9 and 61.9 (3CH_3_ and CH_2_O), 103.2, 119.2, 122.1, 122.9, 128.6, 140.0, 140.8, 147.2 , 147.8, 166.1, 167.2 and 188.2 (Ar-C, 3C=N and C=O). Anal. Calcd for C_16_H_14_Br_2_N_4_O_2_ (454.12): C, 42.32; H, 3.11; N, 12.34. Found: C, 42.33; H, 3.13; N, 12.32.

*Ethyl 3-(2-(2-(6,8-dibromo-2-methylquinazolin-4-yloxy)acetyl) hydr-azono)butanoate* (**8**). A mixture of **4** (10 mmol) and ethyl acetoacetate (10 mmol) in acetic acid (10 mL) was refluxed for 5 h, cooled and the reaction mixture was poured onto ice-water to give a colorless powder which was crystallized from ethanol/acetic acid. Yield 75%; m.p.: 128–129 °C. IR (KBr): 1,735 cm^−1^ (C=O, ester), 1,685 cm^−1^ (C=O, amide) and 1,618 cm^−1^ (C=N); ^1^H-NMR: δ = 1.29 (t, 3H, CH_3_CH_2_), 1.94 (s, 3H, N=C-CH_3_), 2.3 (s, 2H, CH_2_CO), 2.44 (s, 3H, CH_3_, quinazoline), 4.13 (q, 2H, CH_2_CH_3_), 4.63 (s, 2H, OCH_2_CO), 7.95, 8.18 (2s, 2H, Ar-H), 10.68 (s, 1H, NH); ^13^C-NMR: δ = 8.9 (N=CCH_3_), 14.1 (CH_3_CH_2_), 25.8 (CH_3_, quinazoline), 43.5 (CH_2_COO), 61.5 (CH_3_CH_2_O), 69.7 (OCH_2_CO), 133.8, 119.6, 122.3, 123.5, 140.9, 149.5, 153.8, 168.3, 171.1, 173.3 and 181.4 (Ar-C, 3C=N and 2C=O). Anal. Calcd for C_17_H_18_Br_2_N_2_O_4_ (502.16): C, 40.66; H, 3.61; N, 11.16. Found: C, 40.65; H, 3.60; N, 11.17.

*1-(2-(6,8-Dibromo-2-methylquinazolin-4-yloxy)acetyl)-3-methyl-1H-pyrazol-5(4H)-one* (**9**). A solution of 8 (10 mmol) in sodium hydroxide (10%, 20 mL) was boiled under reflux for 6 h, then the reaction mixture was poured onto ice-water and neutralized with dilute HCl. The solid obtained was collected by filtration, washed with water and crystallized from ethanol. Yield 68%; m.p.: 146–147 °C. IR (KBr): 1,685 and 1,668 cm^−1^ (2 C=O, amide); ^1^H-NMR: δ = 1.94 (s, 3H, CH_3_, pyrazole), 2.20 (s, 2H, CH_2_CO, pyrazole), 2.44 (s, 3H, CH_3_, quinazoline), 7.96, 8.19 (2s, 2H, Ar-H). ^13^C-NMR: δ = 16.4 (CH_3_, pyrazole), 25.4 (CH_3_, quinazoline), 42.5 (CH_2_O, pyrazole), 66.7 (OCH_2_O), 113.7, 119.5, 122.1, 123.4, 140.9, 149.5, 159.3, 163.0, 170.6, 171.0 and 181.3 (Ar-C, 2C=O and 3C=N). Anal. Calcd for C_15_H_12_Br_2_N_4_O_3_ (456.09): C, 39.50; H, 2.65; N, 12.28. Found: C, 39.52; H, 2.66; N, 12.26.

### 4.1. General Procedure for the Synthesis of Compounds ***10a-c***

A mixture of **4** (10 mmol) and benzaldehyde derivatives (10 mmol) in absolute ethanol (10 mL) and a few drops of acetic acid was refluxed for 10 h, and the reaction mixture left to cool. The colorless solid was filtered off, and crystallized from ethanol/acetic acid.

*N'-Benzylidine-2-(6,8-dibromo-2-methylquinazolin-4-yloxy)acetohydrazide* (**10a**). Yield 80%; m.p.: 241–242 °C. IR (KBr): 3,285 cm^−1^ (NH), 1,680 cm^−1^ (C=O) and 1,623 cm^−1^ (C=N); ^1^H-NMR: δ = 2.57 (s, 3H, CH_3_), 4.95 (s, 2H, CH_2_O), 7.80–8.38 (m, 7H, Ar-H), 10.2 (s, 1H, N=CH), 11.28 (s, 1H, NH); ^13^C-NMR: δ = 23.1 (CH_3_), 61.5 (OCH_2_CO), 118.6, 119.1, 120.8, 122.2, 127.8, 128.2, 132.5, 139.8, 143.6, 149.5, 155.8, 169.6, 170.3 and 176.8 (Ar-C, 3C=N and C=O). Anal. Calcd for C_18_H_14_Br_2_N_4_O_2_ (478.14): C, 45.22; H, 2.95; N, 11.72. Found: C, 45.21; H, 2.93; N, 11.74.

*N'-(4-Chlorobenzylidine)-2-(6,8-dibromo-2-methylquinazolin-4-yloxy)-acetohydrazide* (**10b**). Yield 85%; m.p.: 239–241 °C. IR (KBr): 3,290 cm^−1^ (NH), 1,685 cm^−1^ (C=O) and 1,628 cm^−1^ (C=N); ^1^H-NMR: δ = 2.57 (s, 3H, CH_3_), 4.94 (s, 2H, CH_2_O), 7.52 (d, 2H, *J* = 8.20 Hz, Ar-H), 7.77 (d, 2H, *J* = 8.45 Hz, Ar-H), 8.15, 8.34 (2s, 2H, Ar-H), 10.20 (s, 1H, N=CH), 11.9 (s, 1H, NH); ^13^C-NMR: δ = 23.2 (CH_3_), 61.8 (OCH_2_CO), 118.6, 119.4, 121.8, 122.7, 128.0, 128.5, 132.7, 139.8, 143.6, 149.8, 156.5, 168.6, 169.8 and 177.8 (Ar-C, 3C=N and C=O). Anal. Calcd for C_18_H_13_Br_2_ClN_4_O_2_ (512.58): C, 42.18; H, 2.56; N, 10.93. Found: C, 42.19; H, 2.58; N, 10.95.

*2-(6,8-Dibromo-2-methylquinazolin-4-yloxy)-N'-(4-methoxyben-zylidine)-acetohydrazide* (**10c**). Yield 84%; m.p.: 245–246 °C. IR (KBr): 3,280 cm^−1^ (NH), 1,685 cm^−1^ (C=O) and 1,618 cm^−1^ (C=N); ^1^H-NMR: δ 2.58 (s, 3H, CH_3_), 3.78 (s, 3H, OCH_3_), 4.95 (s, 2H, OCH_2_CO), 7.32 (d, 2H, *J* = 8.32 Hz, Ar-H), 7.78 (d, 2H, *J* = 8.48 Hz, Ar-H), 8.12, 8.33 (2s, 2H, Ar-H), 10.32 (s,1H, N=CH), 11.85 (s, 1H, NH). Anal. Calcd for C_19_H_16_Br_2_N_4_O_3_ (508.16): C, 44.91; H, 3.17; N, 11.03. Found: C, 44.93; H, 3.18; N, 11.05.

### 4.2. General Procedure for the Synthesis of Compounds ***11a-c***

A mixture of **10a-c** (10 mmol) and thioglycolic acid (10 mmol) in dry pyridine (10 mL) was refluxed for 6 h, cooled, and the reaction mixture was poured onto cold dil. HCl. The solid obtained was filtered off, and crystallized from ethanol.

*2-(6,8-Dibromo-2-methylquinazolin-4-yloxy)-N-(4-oxo-2-phenyl-thiazolidin-3-yl)acetamide* (**11a**). Yield 75%; m.p.: 153–155 °C. IR (KBr): 3,285 cm^−1^ (NH), 1,695 and 1,680 cm^−1^ (2C=O, amide); ^1^H-NMR: δ = 2.59 (s, 3H, CH_3_), 3.67 (s, 3H, CH_2_S), 4.89 (s, 2H, OCH_2_CO), 5.82 (s, 1H, CHPh), 7.82–8.52 (m, 7H, Ar-H), 10.40 (br, 1H, NH). Anal. Calcd for C_20_H_16_Br_2_N_4_O_3_S (552.24): C, 43.50; H, 2.92; N, 10.15. Found: C, 43.56; H, 2.85; N, 10.28. 

*N-(2-(4-Chlorophenyl)-4-oxothiazolidin-3-yl)-2-(6,8-dibromo-2-methylquinazolin-4-yloxy)acetamide* (**11b**). Yield 78%; m.p.: 158–160 °C. IR (KBr): 3,295 cm^−1^ (NH), 1,690, 1,682 cm^−1^ (2C=O, amide); ^1^H-NMR: δ = 2.59 (s, 3H, CH_3_), 3.69 (s, 3H, CH_2_S), 4.89 (s, 2H, OCH_2_CO), 5.79 (s, 1H, CHPh), 7.53 (d, 2H, *J* = 8.20 Hz, Ar-H), 7.78 (d, 2H, *J* = 8.28 Hz, Ar-H), 8.11 and 8.24 (2s, 2H, Ar-H), 10.50 (br, 1H, NH); ^13^C-NMR: δ = 23.3 (CH_3_), 39.4, 54.7, 61.9 (CH_2_S, CHPh and OCH_2_CO), 119.1, 122.2, 123.0, 128.7, 128.8, 134.8, 136.3, 140.1, 144.0, 148.5, 156.5, 168.6, 168.8, 169.6 and 176.8 (Ar-C, 2C=N and 2C=O). Anal. Calcd for C_20_H_15_Br_2_ClN_4_O_3_S (586.68): C, 40.94; H, 2.58; N, 9.55. Found: C, 40.93; H, 2.57; N, 9.55.

*2-(6,8-Dibromo-2-methylquinazolin-4-yloxy)-N-(2-(4-methoxyph-enyl)4-oxothiazolidin-3-yl)acetamide* (**11c**). Yield 85%; m.p.: 168–170 °C. IR (KBr): 3,310 cm^−1^ (NH), 1,695 and 1,685 cm^−1^ (2C=O, amide); ^1^H-NMR: δ = 2.58 (s, 3H, CH_3_), 3.67 (s, 3H, CH_2_S), 3.87 (s, 3H, OCH_3_), 4.98 (s, 2H, OCH_2_CO), 5.92 (s,1H, CHPh), 7.33 (d, 2H, *J *= 8.30 Hz, Ar-H), 7.78 (d, 2H, *J* = 8.62 Hz, Ar-H), 8.15 and 8.36 (2s, 2H, Ar-H), 11.90 (br, 1H, NH). Anal. Calcd for C_21_H_18_Br_2_N_4_O_4_S (582.27): C, 43.32; H, 3.12; N, 9.62. Found: C, 43.33; H, 3.15; N, 9.62.

*2-(2-(6,8-Dibromo-2-methylquinazolin-4-yloxy)acetyl)-N-phenylh-ydrazineecarbothioamide *(**12a**). A mixture of **4** (10 mmol), and phenyl isothiocyanate (10 mmol) in dry dioxane (20 mL) was refluxed for 4 h and cooled. The solid obtained was filtered off, dried and crystallized from ethanol/acetic acid. Yield 75%; m.p.: 219–220 °C. IR (KBr): 3,280 cm^−1^ (3NH), 1,685 cm^−1^ (C=O, amide) and 1,320 (C=S); ^1^H-NMR: δ = 2.55 (s, 3H, CH_3_), 4.92 (s, 2H, OCH_2_CO), 7.12 (s, 1H, NHCS), 7.23–8.32 (m, 7H, Ar-H), 10.6 (s, 1H, NHCO), 12.52 (s, 1H, NH). Anal. Calcd for C_18_H_15_Br_2_N_5_O_2_S (525.22): C, 41.16; H, 2.88; N, 13.33. Found: C, 41.16; H, 2.85; N, 13.32.

*N-Cyclohexyl-2-(2-(6,8-dibromo-2-methylquinazolin-4-yloxy)ac-etyl)hydrazinecarbothioamide* (**12b**). A mixture of **4** (10 mmol) cyclohexyl isothiocyanate (10 mmol) in dry dioxane (20 mL) was refluxed for 4 h, then left to cool. The solid obtained was filtered off, dried and crystallized from ethanol/acetic acid. Yield 72%; m.p.: 228–230 °C. IR (KBr): 3,290 cm^−1^ (3NH), 1,680 cm^−1^ (C=O, amide) and 1,335 cm^−1^ (C=S); ^1^H-NMR: δ = 1.24–1.76–2.02 (m, 10H, 5CH_2_, cyclohexane), 2.57 (s, 3H, CH_3_), 3.20 (m, 1H, C_1_-cyclohexane), 4.93 (s, 2H, OCH_2_CO), 7.20 (s, 2H, 2NH), 8.15–8.38 (2s, 2H, Ar-H), 10.6 (br, 1H,NHCO). ^13^C-NMR: δ = 22.6, 23.2, 27.1, 32.4, 51.2 (cyclohexane and CH_3_), 61.8 (OCH_2_CO), 113.7, 119.5, 122.1, 123.4, 140.9, 149.5, 165.8, 166.2, 176.5 and 185.8 (Ar-C, 2C=N, C=O and C=S). Anal. Calcd for C_18_H_21_Br_2_N_5_O_2_S (531.26): C, 40.69; H, 3.98; N, 13.18. Found: C, 40.68; H, 3.99; N, 13.19.

### 4.3. General Procedure for the Synthesis of ***13a,b***

Compounds **12a,b** (1.0 g) were refluxed in 5% Na_2_CO_3_ solution (10 mL) for 5 h. The mixture was left to cool, filtered, and the filtrate was acidified with dil. HCl. The solid obtained was filtered off, and crystallized from ethanol to give colorless crystals.

*5-((6,8-Dibromo-2-methylquinazolin-4-yloxy)methyl)-4-phenyl-4H-1,2,4-triazole-3-thiol* (**13a**). Yield 65%; m.p.: 198–200 °C. IR (KBr): 2,565 cm^−1^ (SH) and 1,625 cm^−1^ (C=N); ^1^H-NMR: δ = 2.56 (s, 3H, CH_3_), 4.95 (s, 2H, OCH_2_), 7.39–8.45 (m, 7H, Ar-H), 13.35 (s, 1H, SH); ^13^C-NMR: δ = 23.5 (CH_3_), 61.8 (OCH_2_), 113.7, 119.5, 122.1, 123.4, 126.2, 128.0, 129.2, 129.8, 140.9, 148.5, 149.1, 163.0, 168.5 and 176.1 (Ar-C and 4C=N). Anal. Calcd for C_18_H_13_Br_2_N_5_OS (507.20): C, 42.62; H, 2.58; N, 13.81. Found: C, 42.44; H, 2.56; N, 13.82.

*4-Cyclohexyl-5-((6,8-dibromo-2-methylquinazolin-4-yloxy)me-thyl)-4H-1,2,4-triazole-3-thiol* (**13b**). Yield 63%; m.p.: 204–205 °C. IR (KBr): 2,572 cm^−1^ (SH) and 1,618 cm^−1^ (C=N); ^1^H-NMR: δ = 1.22–1.95 (m, 10H, 5CH_2_, cyclohexane), 2.51(s, 3H, CH_3_), 3.28 (m, 1H, C_1_-cyclohexane), 4.95 (s, 2H, CH_2_O), 8.15 and 8.38 (2s, 2H, Ar-H), 13.38 (s, 1H, SH). Anal. Calcd for C_18_H_19_Br_2_N_5_OS (513.25): C, 42.12; H, 3.73; N, 13.65. Found: C, 42.14; H, 3.75; N, 13.64.

### 4.4. General Procedure for the Synthesis of ***14a,b***

Compounds **12a,b** (1.0 g) were stirred at 5 °C for 1 h in conc. H_2_SO_4_ (20 mL) then the reaction mixture was left to stir at room temperature for another 15 h. The reaction mixture was poured onto ice-water; the solid obtained was filtered off, washed with water, dried and crystallized from ethanol/acetic acid.

*5-((6,8-Dibromo-2-methylquinazolin-4-yloxy)methyl)-N-phenyl-1,3,4-thiadiazol-2-amine* (**14a**). Yield 52%; m.p.: 180–182 °C. IR (KBr): 3,280 cm^−1^ (NH) and 1,625 cm^−1^ (C=N); ^1^H-NMR: δ = 2.56 (s, 3H, CH_3_), 4.01 (s, 1H, NH), 4.85 (s, 2H, CH_2_O), 6.50–8.38 (m, 7H, Ar-H); ^13^C-NMR: δ 23.6 (CH_3_), 61.9 (CH_2_O), 113.8, 119.5, 122.0, 123.5, 126.4, 128.0, 129.2, 129.8, 140.9, 147.5, 148.8, 164.2, 169.5 and 177.0 (Ar-C and 4C=N). Anal. Calcd for C_18_H_13_Br_2_N_5_OS (507.20): C, 42.62; H, 2.58; N, 13.81. Found: C, 42.60; H, 2.59; N, 13.82.

*N-Cyclohexyl-5-((6,8-dibromo-2-methylquinazolin-4-yloxy)me-thyl)-1,3,4-thiadiazol-2-amine* (**14b**). Yield 55%; m.p.: 185–186 °C. IR (KBr): 3,280 cm^−1^ (NH) and 1,628 cm^−1^ (C=N). ^1^H-NMR: δ = 1.22–1.98 (m, 10H, 5CH_2_, cyclohexane), 2.56 (s, 3H, CH_3_), 3.29 (m, 1H, C_1_-cyclohexane), 4.08 (s, 1H, NH), 4.98 (s, 2H, CH_2_O), 8.13–8.42 (2s, 2H, Ar-H). Anal. Calcd for C_18_H_19_Br_2_N_5_OS (513.25): C, 42.12; H, 3.73; N, 13.65. Found: C, 42.13; H, 3.73; N, 13.64.

*3-((6,8-Dibromo-2-methylquinazolin-4-yloxy)methyl-5H-[1,2,4]-triazolo[3,4-a]isoindol-5-one* (**15**). A mixture of **4** (10 mmol) and phthalimide (10 mmol) was fused in an oil bath at 180 °C for 3 h. After cooling the reaction mixture was poured onto ice-cold HCl. The solid obtained was filtered off, washed with water and crystallized from ethanol/acetic acid to give a pale brown powder. Yield 81%; m.p.: 285–286 °C. IR (KBr): 1,680 cm^−1^ (C=O) and 1,628 cm^−1^ (C=N); ^1^H-NMR: δ = 2.57 (s, 3H, CH_3_), 4.98 (s, 2H, CH_2_O), 7.50–8.35 (m, 6H, Ar-H). ^13^C-NMR: δ = 23.2 (CH_3_), 61.89 (CH_2_O), 114.0, 119.8, 122.1, 123.8, 127.6, 129.2, 130.5, 134.4, 135.2, 137.7, 140.9, 149.5, 150.2, 162.0, 167.2, 176.8 and 185.8 (Ar-C, 4C=N and C=O). Anal. Calcd for C_19_H_11_Br_2_N_5_O_2_ (501.13): C, 45.54; H, 2.21; N, 13.98. Found: C, 45.53; H, 2.23; N, 13.97.

## 5. Conclusions

We have used simple and convenient methods with simple work up and producing clean products for the synthesis of novel heterocycles such as pyrazolone, pyrazole, thiazole, 1,2,4-triazole and 1,3,4-thiadiazole moieties with 6,8-dibromo-2-methylquinazolin-4(3*H*)-one as substituent from 2-(6,8-dibromo-2-methylquinazolin-4-yloxy)acetohydrazide. All the compounds tested for analgesic activity showed higher activity than valdecoxib(g) used as a reference drug.
